# Evaluating the electronic tuberculosis register surveillance system in Eden District, Western Cape, South Africa, 2015

**DOI:** 10.1080/16549716.2017.1360560

**Published:** 2017-08-29

**Authors:** Mandla Mlotshwa, Sandra Smit, Seymour Williams, Carl Reddy, Andrew Medina-Marino

**Affiliations:** ^a^ South African Field Epidemiology Training Programme, National Institute for Communicable Diseases, Johannesburg, South Africa; ^b^ Epidemiology Research Unit, Foundation for Professional Development, Pretoria, South Africa; ^c^ School of Health System and Public Health, University of Pretoria, Pretoria, South Africa; ^d^ Department of Health, Eden District, Western Cape Province, George, South Africa; ^e^ US Centers for Disease Control and Prevention, Pretoria, South Africa; ^f^ Division of Global Health Protection, Center for Global Health, Centers for Disease Control and Prevention, Atlanta, USA

**Keywords:** Tuberculosis, surveillance system, ETR.Net, TB control programs, South Africa

## Abstract

**Background**: Tuberculosis (TB) surveillance data are crucial to the effectiveness of National TB Control Programs. In South Africa, few surveillance system evaluations have been undertaken to provide a rigorous assessment of the platform from which the national and district health systems draws data to inform programs and policies.

**Objective**: Evaluate the attributes of Eden District’s TB surveillance system, Western Cape Province, South Africa.

**Methods**: Data quality, sensitivity and positive predictive value were assessed using secondary data from 40,033 TB cases entered in Eden District’s ETR.Net from 2007 to 2013, and 79 purposively selected TB Blue Cards (TBCs), a medical patient file and source document for data entered into ETR.Net. Simplicity, flexibility, acceptability, stability and usefulness of the ETR.Net were assessed qualitatively through interviews with TB nurses, information health officers, sub-district and district coordinators involved in the TB surveillance.

**Results**: TB surveillance system stakeholders report that Eden District’s ETR.Net system was simple, acceptable, flexible and stable, and achieves its objective of informing TB control program, policies and activities. Data were less complete in the ETR.Net (66–100%) than in the TBCs (76–100%), and concordant for most variables except pre-treatment smear results, antiretroviral therapy (ART) and treatment outcome. The sensitivity of recorded variables in ETR.Net was 98% for gender, 97% for patient category, 93% for ART, 92% for treatment outcome and 90% for pre-treatment smear grading.

**Conclusions**: Our results reveal that the system provides useful information to guide TB control program activities in Eden District. However, urgent attention is needed to address gaps in clinical recording on the TBC and data capturing into the ETR.Net system. We recommend continuous training and support of TB personnel involved with TB care, management and surveillance on TB data recording into the TBCs and ETR.Net as well as the implementation of a well-structured quality control and assurance system.

## Background

South Africa has the third highest tuberculosis (TB) incidence rate (860 cases per 100,000 population) in the world [1], and an epidemic fueled by human immunodeficiency virus (HIV) [2]. Given the high burden of TB in South Africa, the need to manage an effective TB surveillance system to plan and evaluate TB control programs remains a critical goal. Surveillance data are essential to guide planning and policy decision making. These data are also essential to ensure that South Africa achieves the United Nations Sustainable Development Goals of reducing TB incidence by 80% and mortality by 90% compared with their 2015 baseline by 2030 [3,4].

In South Africa, the Electronic TB register (ETR.Net) surveillance system has been operational since 1995 and provides the National TB Control Program (NTCP) with information on key indicators essential to guide and inform public health action and TB programmatic activities [5,6]. Primary data for ETR.Net is based on the TB Blue Card (TBC), the primary medical file of TB patients. The TBC is transcribed into the TB Register, a paper-based register, which is used to enter information into ETR.Net at the sub-district and district levels. Several challenges and deficiencies with the recording and reporting in ETR.Net surveillance system have been identified, including poor data quality, under-reporting of TB cases, multiple data entry, inadequate number of data capturers and lack of comprehensive understanding of the importance and utility of accurate and reliable data [7–9]. These challenges lead to an underestimation of true burden of TB, poor implementation of appropriate control strategies and misallocation of resources. A recent review of the TB surveillance data in three of South Africa’s nine provinces revealed that 34% of smear positive TB cases recorded in a clinic’s paper-based TB Suspect Register were not recorded in the clinic’s TB Register, and did not have a TBC opened for that patient [9]; the TB Suspect Register is used to track individuals with signs and symptoms of TB that provided a sputum specimen for diagnostic testing. This reflects a missed opportunity for the TB control program to enroll these patients into TB care and disease management, and to mitigate the spread of TB in the communities. Poor data quality of clinical variables used by the NTCP to integrate the TB and HIV programmatic data in the ETR.Net has also been reported [10]. This also hinders the implementation of appropriate prevention and control strategies for HIV and TB co-infection.

In line with World Health Organization (WHO) recommendations, it is critical to periodically evaluate TB surveillance systems in order to identify challenges and barriers to complete and accurate data essential to TB management and control activities. To ensure the NTCP’s objectives are being met, we evaluated Eden District’s TB surveillance system using the US Centers for Disease Control and Prevention’s (CDC’s) guidelines for evaluating public health surveillance systems [11].

## Methods

### Study design

A quantitative and qualitative evaluation of the Eden District, Western Cape Province, ETR.Net surveillance system was conducted from May to June 2014 and March 2015 using CDC guidelines for evaluating public health surveillance systems as described in Supplementary Table 1 [11]. We performed data review of ETR.Net TB source and conducted semi-structured interviews with key clinic and district-based personnel involved with TB care, management and surveillance.

**Table 1.** Stakeholders interviewed and attributes assessed during an evaluation TB surveillance system in Eden District, 2015.

Stakeholders	Number participated	Attributes assessed
TB nurses	3	Acceptability, simplicity
Information health officers or data capturers	2*	Acceptability, flexibility, stability
Sub-district HAST coordinators	3	Usefulness, acceptability, simplicity, flexibility, stability
District HAST coordinators	1	Usefulness, simplicity, acceptability

*Sub-district coordinator in Knysna was interviewed as health information officer as she captures data in the ETR.Net in Knysna sub-district.

### Study setting and health facility selection

**Figure 1.**
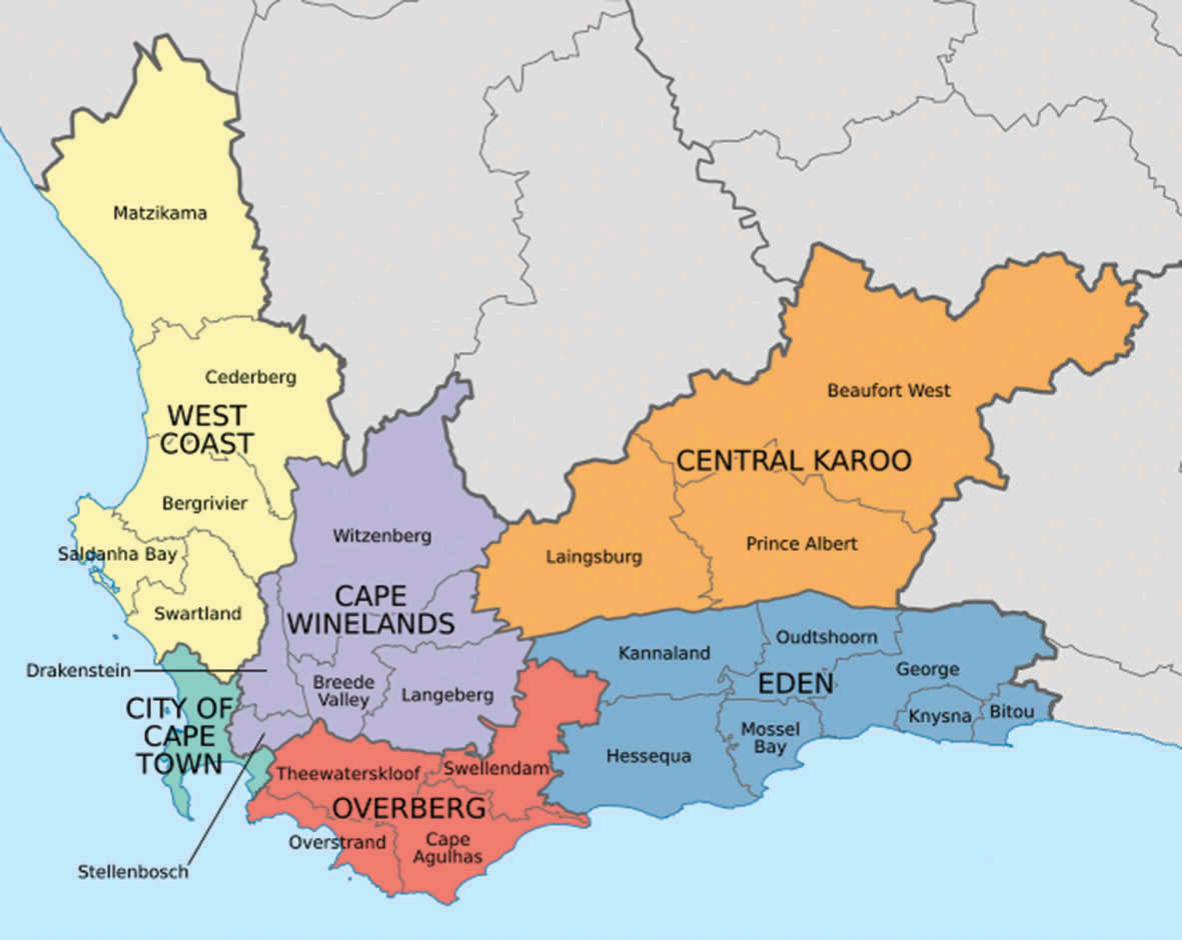
District and sub-district level map of Western Cape Province, South Africa [12].

Eden District is one of six districts in Western Cape Province, South Africa (Figure 1). Eden has an estimated population of 585,833 people with a population density of 25.1 persons per km^2^ [13]. Eden is divided into seven sub-districts: Bitou, George, Hessequa, Knysna, Kannaland, Mossel Bay and Oudtshoorn (Figure 1). The district is predominantly urban and its main economic activities are finance, business services, manufacturing, wholesale trading, catering and accommodation, general government services, transport and agriculture [13]. The TB control program is based on the network of its sub-districts’ primary and community health clinics, which offer TB screening and treatment based on South African standard guidelines [14]. In 2013, Eden’s incidence of TB was 806 per 100,000 population, which was above the incidence rate of 711 per 100,000 population in Western Cape Province [15].

Eighteen primary health clinic (PHC) facilities were conveniently selected from among the district’s 67 PHCs based on a previous investigation [15]. From the 18 PHCs, a random selection of 79 TBCs was performed based on the 602 pulmonary TB patients that had not converted to TB smear negative at the end of the intensive phase of treatment from 2010 to 2013. For qualitative evaluation of Eden’s District ETR.Net, we purposively selected three sub-districts (Bitou, Knysna and George) for inclusion based on their large catchment areas and high incidence rates of TB–HIV co-infection.

### Data source and collection

#### Quantitative data

Primary data sources included TBCs and Eden District’s ETR.Net database. The TBC is a patient’s primary medical record and is the source document from which ETR.Net data is collected. ETR.Net is the electronic database which records TBC data from TB patients that initiated treatment; TB-infected individuals that do not initiate TB treatment do not have TBCs, and thus are not recorded in ETR.Net. ETR.Net is used by all 52 of South Africa’s Health Districts and the NTCP to monitor and evaluate key TB performance indicators.

**Figure 2.**
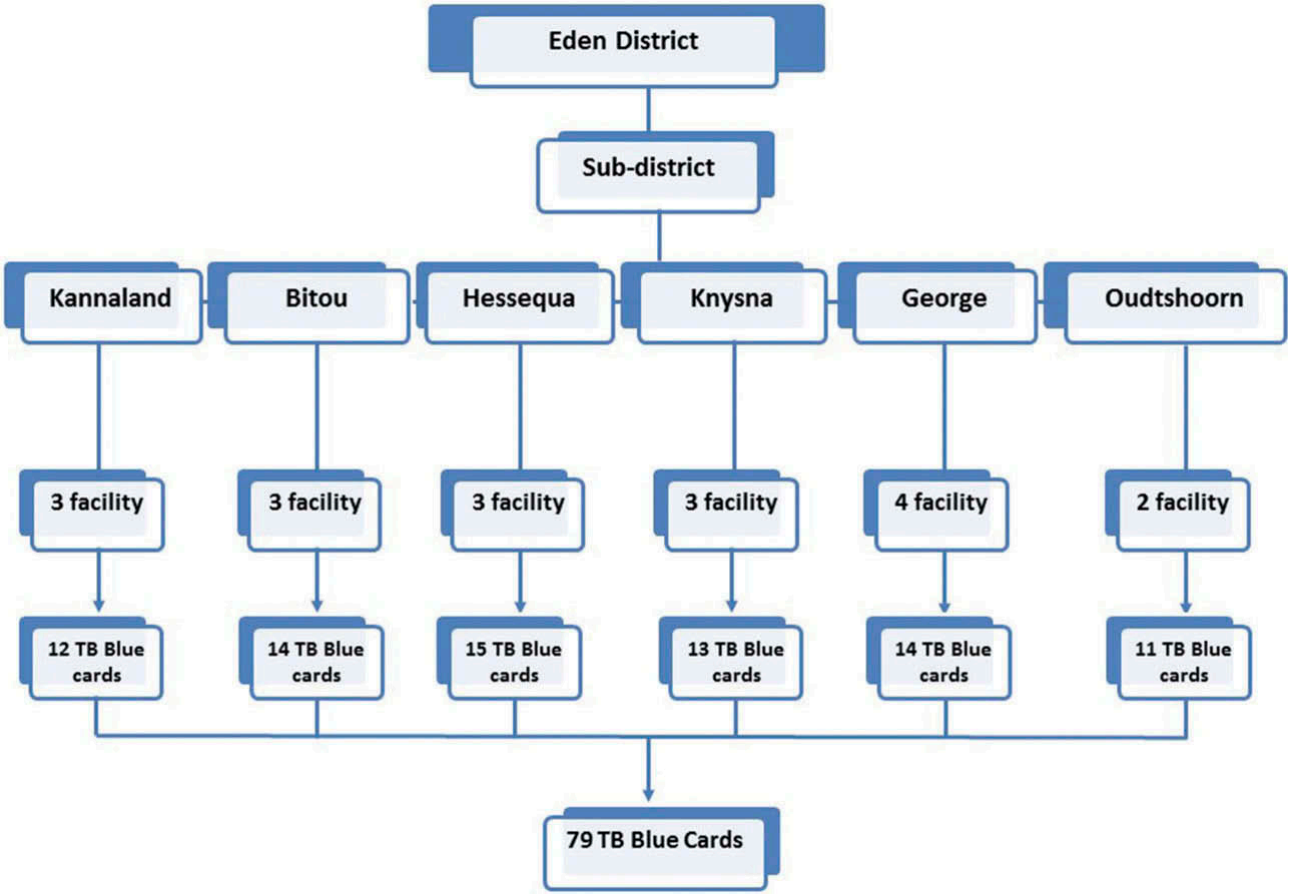
Tuberculosis evaluation sites included in the data review in Eden District, Western Cape, 2014.

TBCs from selected facilities were abstracted to assess the validity and completeness of TB surveillance data (i.e. completeness, concordance, sensitivity and positive predictive value [PPV]) recorded in ETR.Net (Figure 2). Data variables used to assess data quality included: date of birth, gender, patient category (new, retreatment after failure, retreatment after default, other retreatment, relapse), TB classification (pulmonary or extra-pulmonary), treatment outcome, treatment outcome date, HIV status, CD4 count, treatment start date, pre-treatment smear results and grading, on antiretroviral therapy (ART) and cotrimoxazole preventive therapy (CPT).

#### Qualitative data

An operational assessment of key domains and attributes of the ETR.Net surveillance system, including usefulness, simplicity, acceptability, stability and flexibility, was performed using semi-structured, face-to-face interviews with clinic, sub-district and district-level key informants (Table 1) [8]. Key informants were selected basis on their involvement with key aspects of surveillance activities. We defined key domains and attributes of the TB surveillance system as follows: (i) *usefulness* – how was the information collected in the TB surveillance used for public health action; (ii) *simplicity* – ease of use and operational structure of TB surveillance system; (iii) *acceptability* – willingness of district TB program staff to participate in the reporting and recording of data in ETR.Net; (iv) *stability* – the ability of the TB surveillance to collect, manage and provide data without interruption or failure; and (v) *flexibility* – how the TB surveillance accommodates changes in information needs or operating conditions with minimal additional time, personnel and cost. Detailed notes were taken during interviews, and open coding of content was performed to ascertain both positive and negative experiences and perspectives related to the key domains and attributes of the system [16].

### Statistical analysis

Data were captured in Epi-Info7 (CDC, http://www.cdc.gov/epiinfo/) and analyzed using STATA 13.0 (Stata Corporation, College Station, Texas, USA). Data quality was evaluated by assessing the completeness, concordance, sensitivity and PPV of reported values in the ETR.Net. Completeness of data was calculated as the total number of values available for each variable divided by the total number of records. Data concordance, a measurement of agreement, between TBCs and ETR.Net was calculated using Cohen’s Kappa (κ) coefficient for categorical variables and intraclass correlation coefficient (ICC) for continuous variables. We interpreted the κ values as follows: 0.81–1 as near perfect agreement, 0.61–0.80 as substantial agreement, 0.41–0.60 as moderate agreement, 0.21–0.40 as fair agreement, <0.21 as poor agreement and −1 as perfect disagreement. An ICC value of >0.75 represents excellent reliability, 0.40–0.75 represents fair to good reliability and <0.40 represents poor reliability. Sensitivity and PPV of variables in the ETR.Net were computed using a *diagt* command in STATA. Sensitivity was defined as the probability of having a positive value in the ETR.Net given a positive value in the TBC. PPV was defined as the probability of a documented positive value in the TBC given a positive value in the ETR.Net.

### Ethical consideration

Permission to conduct this evaluation was obtained from Eden District and the Western Cape Provincial Department of Health. This evaluation was designated as a non-research program evaluation by the Foundation for Professional Development Research Ethics Committee. All data collected through record reviews were anonymized and written informed consent was obtained from all key stakeholders interviewed. This included the purpose of the study, what is expected for participating in the study, the benefits of the study, the right of the participants, confidentiality and consent statement.

## Results

### Description of the system

The data flow and reporting structure of the TB surveillance system is described in Figure 2. Those screened and suspected of having TB are first entered into a facility’s TB Suspect Register. Upon confirmation of TB disease, basic information from the TB Suspect Register is transferred into the facility’s TBCs. Key information from the TBC is then transferred into the facility’s TB Register. TB Registers are then sent to or collected by the sub-district TB program, and TB Register data are entered directly into the ETR.Net by health information officers. At district level, ETR.Net files collected from sub-districts are collated, merged and submitted to the provincial TB office. Once merged at a provincial level, TB data are then sent to the NTCP where national data are analyzed and annual reports developed. Such reports are then shared with the WHO and TB program staff at provincial, district, sub-district and facility levels.

## Quantitative results

### Data quality

Of the 14 variables selected for comparison between TBCs and ETR.Net, 5/14 (36%) TBC variables had a 95–100% completion rate compared with 9/14 (64%) of ETR.Net variables (Table 2). Gender, HIV status, patient category, disease classification and treatment start date variables were completed 95–100% of the time in both TBCs and ETR.Net (Table 2). When comparing completeness of data between TBC and ETR.Net, 29% of date of birth information, 24% of treatment outcome and 12% of treatment outcome date were lost during data transfer (Table 2). Across the two data sources, concordance was good (range 0.89–0.99) for (i) baseline CD4 count (ICC = 0.99), (ii) date of birth or Age (ICC = 0.96), (iii) gender (kappa = 0.89) and (iv) HIV status (kappa = 0.89). However, concordance was poor (range 0.12–0.28) for (i) pre-treatment smear results (kappa = 0.28), (ii) on ART (kappa = 0.24) and (iii) TB treatment outcomes (kappa = 0.12).

**Table 2.** Completeness (%) and concordance of selected variables in the TB Blue Cards and ETR.Net in Eden District, 2015.

		Completeness (%)					
	Total	TB blue card		ETR.Net		Concordance	
Variable	*N*	*n*	%	*n*	%	Agreement (%)	κ (95% CI)
^#^Baseline CD4 count	16	15	94	13	82	85	0.99 (0.87–0.96)
^#^Date of birth or Age	79	75	95	52	66	91	0.96 (0.94–0.98)
Gender	79	78	99	79	100	95	0.89 (0.78–0.99)
HIV status	79	78	99	76	96	96	0.89 (0.87–0.96)
Pre-treatment smear grading	79	61	77	77	97	83	0.68 (0.51–0.80)
Patient category	79	77	97	79	100	82	0.67 (0.57–0.86)
Pre-treatment smear results	79	60	78	78	99	75	0.28 (0.16–0.29)
On ART	11	8	73	10	91	59	0.24 (0.07–0.49)
Treatment outcome	79	71	90	52	66	47	0.12 (0.00–0.14)
Disease classification	79	77	97	79	100	98	0.000
On CPT	8	6	75	8	100	65	−0.097
Treatment start date*	79	77	97	79	100	73	-
Treatment outcome date*	79	62	78	52	66	67	-
Pre-treatment smear date*	79	60	76	78	99	83	-

# Intraclass correlation coefficient (ICC) was used for continuous variable; κ represent kappa statistics for categorical variables. Zero or negative kappa mean no agreement. *n* and % represent the numbers and percentages of TBC and ETR.Net variables analyzed. * ICC for dates were omitted on Stata because of unbalance data.

### Sensitivity and predictive value of reported positive value

The estimated sensitivity of reported values in the ETR.Net ranged from 90% to 98% for gender, patient category, on ART, treatment outcome and pre-treatment smear grading, and 75–79% for HIV status, pre-treatment smear results and CPT (Table 3). All variables except ‘on ART’ (87%, 95% CI: 62–98) had a PPV of 92–99%, indicating that greater than 90% of these variables documented in the TBCs were reported into ETR.Net (Table 3).

**Table 3.** Sensitivity (%) and positive predictive value (%) of selected reported variables in the ETR.Net in Eden District, 2015.

Variable	Sensitivity of reported value		Positive predictive value	
	(%)	95% CI	(%)	95% CI
Gender	98	89–100	94	83.5–99
Patient category	97	91–100	97	91–100
On ART	93	68–100	87	62–98
Treatment outcome	92	84–97	99	92–100
Pre-treatment smear grading	90	76–97	92	79–98
HIV status	79	68–87	97	89–100
Pre-treatment smear results	76	65–85	98	91–100
On CPT	75	47–93	92	64–100

## Qualitative analysis of key attributes of the TB surveillance system

### Objectives of the system

Sub-district coordinators noted that they used ETR.Net to generate reports on TB cases, sputum smear conversion and TB treatment outcomes. They also acknowledged other benefits of ETR.Net: ‘As part of HIV and TB service integration, we use the ETR.Net to evaluate and report on basic data relating to HIV status of TB patients and ART initiation of those with co-infection’ (sub-district coordinator).

### Usefulness

Sub-district and district HIV/AIDS, STIs and TB (HAST) coordinators spoke of how useful the ETR.Net was:

Interviewer: ‘Do you think the information contained in the ETR.Net is adequate to help the sub-district and district to manage the performance of TB control program at the health facilities?’

District coordinator: ‘Yes, reports generated from the ETR.Net are useful to inform and guide resource allocation at the facilities.’

Sub-district coordinator: ‘We can also track the burden of TB at the sub-districts, monitor clinic’s catchment areas with high burden and TB case trends by age, sex HIV status, patient category, health facilities and sub-districts.’

### Acceptability

Overall, eight of nine (88%) respondents (Table 1) reported being satisfied with the reporting and recording of data, and the objectives of the TB surveillance system. At the health facilities TB nurses acknowledged the acceptability of TBCs and TB register:

Interviewer: ‘Do you find it easy to transfer information from a TBCs into a TB register by yourself?’

TB nurse: ‘I think it is a lot easier for us to transfer information and in fact information on the TBCs are similar to those contained on the TB register except that there are no detail on treatment doses, clinics note and household contacts on TB register.’

At the sub-district level, health information officers and HAST coordinators also spoke of the willingness to use the ETR.Net:

Interviewer: ‘Do you find it easy to enter data into the ETR.Net?’

Health information officer: ‘When I started using the ETR.Net it wasn’t easy at all but with training and working on the system on a daily basis, it is much easier to enter information on the system and to generate reports for the health facilities.’

### Simplicity

**Figure 3.**
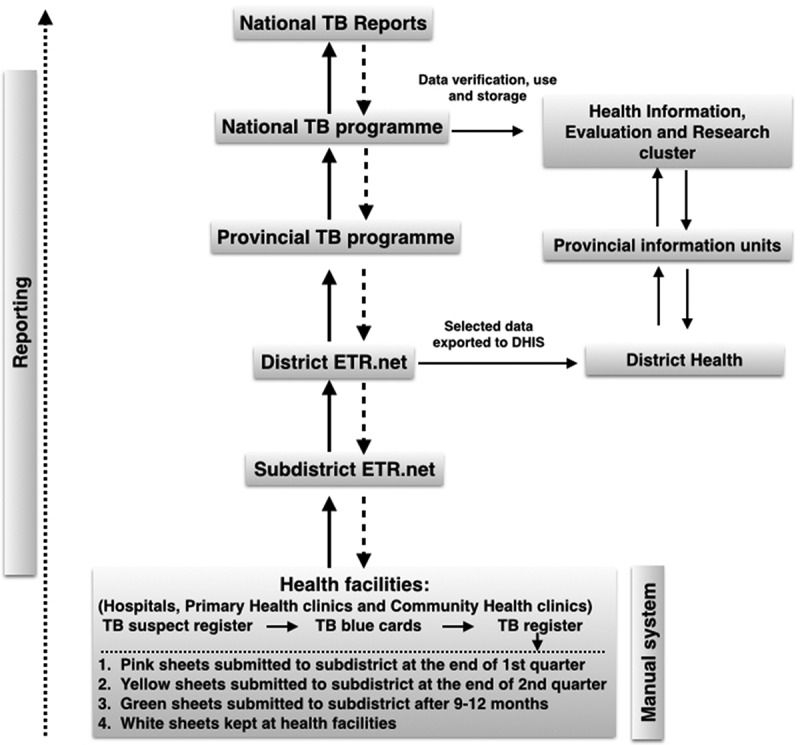
The electronic TB register surveillance system data flow and reporting in South Africa.

The TB surveillance system has a simple data flow structure and reporting scheme as illustrated in Figure 3. The majority of coordinators reported that the ETR.Net, with its user guide, was straightforward and easy to use. Health information officers also acknowledged that ETR.Net has a ‘friendly’ user interface for both data entry and report generation, and provides clear guidance and examples for how to generate reports. Two-thirds of the facility-based TB nurses found that instructions for entering information in the TBC and TB register were self-explanatory and not difficult.

### Stability

Health information officers and sub-district coordinators spoke of how they experience interruption and downtimes on the ETR.Net:

Interviewer: ‘Were there any unscheduled outage and downtimes in the ETR.Net, where data was not captured, analyzed and submitted to the facilities?’

Sub-district coordinator: ‘Yes, at times there were periodic downtimes of 2–6 hours in a week in 2014 where we couldn’t use the system and this was due to the installation of ETR.Net v2.’

Health information officer: ‘We had some problems with login and random freeze of the system due updates and software changes.’

### Flexibility

Prior to the introduction of GeneXpert testing and results capture on ETR.Net, it was not possible to distinguish whether TB patients tested positive using GeneXpert or culture, as during this time GeneXpert results were entered in the culture results section. Since this was not optimal for flexibility, the system was later adapted to accommodate the changing information needs for the revised WHO algorithms for TB diagnosis to include GeneXpert testing. Subsequent to this, in 2014, more improvements were made to ETR.Net to include enhanced security features, locking of records, data migration tool and data exchange with other electronic health information systems (e.g. the district health information system, three interlinked electronic register and EDRWeb). However, despite these improvements, sub-district HAST coordinators expressed concerns that ETR.Net was not networked and lacked unique TB patient IDs.

## Discussions

One of the key findings in this evaluation was the perceived usefulness of the TB surveillance system and the high level of acceptability among its stakeholders. Stakeholders were very satisfied with information gathered through the surveillance system and its use for public health program planning, implementation and resource allocation. Given the importance of surveillance data to the monitoring of TB programmatic indicators, it is critical that health facility and TB program staff ensure quality and completeness of surveillance system data. Our findings of usefulness and acceptability of the system in Eden District is encouraging, and demonstrate a direct engagement of stakeholders with TB surveillance system to inform TB programmatic activities and policies. Continuous engagement and support of TB staff is essential to improve the understanding and objectives of TB surveillance, and to identify challenges and barriers critical in the management and control of TB in the district.

Our evaluation also showed that the system was simple, stable and flexible. Most TB clinical staff found the surveillance data collection tools easy to use, while TB program managers felt that data collection tools collected appropriate programmatic indicators to inform programs and resource allocation. While there was periodic downtime of the system due to regular updates and random system freezes, stakeholders believed the system was stable and adaptable to changing information needs; changes were made to enhance security features and accommodate the revised WHO algorithms for GeneXpert testing. However, stakeholders also suggested that additional efforts are needed to network the system and to include a unique ID for TB patients. Without a unique patient ID, it is difficult to update patient information of those that move between or transfer clinics, thus impacting patient tracking, planning and allocation of resources in the sub-districts.

Data discrepancies were found within the TB surveillance tools. Specifically, date of birth, age, treatment outcomes, baseline CD4 counts and on ART were not adequately completed in the ETR.Net; data completeness for these key variables ranged from 66% to 89%. Sub-optimal data quality can undermine the ability of the NTCP to properly evaluate and plan for TB control program activities in the district, and the utility of the TB surveillance system to inform public health actions and policies. Sound, reliable data quality is also critical to instill confidence of stakeholders in the performance of the surveillance system. We attribute the findings of sub-optimal data quality in Eden to the lack of dedicated sub-district health information officers, inadequate computers, staff training, and standard operating procedures for tracking updates and changes to patient information in the ETR.Net.

Furthermore, it is evident that there is a need for TB program managers at the sub-district and district levels to implement structured quality control and assurance processes to improve the integrity of data collected in the TB surveillance system. Continuous training of personnel involved with data quality at different levels of the TB surveillance system is also an important mechanism to engage them on the purpose and uses of the TB surveillance data to achieve the objective of the South African NTCP.

Despite this, our findings demonstrated a good sensitivity of reported values in the ETR.Net for gender, patient category, on ART, pre-treatment smear grading and treatment outcomes. In addition, we report good PPV for gender, patient category, treatment outcome, HIV status, pre-treatment smear results and CPT variables ranging from 92% to 99%, indicating that less than 10% of these variables were missing in the ETR.Net. Such findings demonstrate the robustness of the TB surveillance system to capture these key variables which are critical to the performance of the TB surveillance system and control of TB in the district. To our knowledge, this first study to report on the sensitivity and PPV of recorded variables on the district’s South African TB surveillance system. Additional data might be needed to establish whether this finding is generalizable to other districts in the Western Cape Province or in South Africa as whole.

### Limitations of the study

This evaluation has several limitations. The scope of the evaluation was limited to Eden District and is not generalizable to other districts in Western Cape Province. Our data review was restricted to specifically to TBCs and ETR.Net, not the processes or procedures associated with the flow and transfer of data from TBCs to the TB register, and finally to ETR.Net. Recent evaluation of TB data sources in South Africa suggest that information is often lost when transferred from TBCs to TB register and from TB register to ETR.Net [14].

## Conclusions

Despite its limitations, ETR.Net provides valuable surveillance data and information for Eden District. ERT.Net is simple, acceptable, flexible, stable and useful in achieving the objectives of guiding the district’s TB control program and activities. To strengthen the ETR.Net surveillance system we recommend the following: (i) implementation of a quality assurance mechanism to improve data quality, (ii) continuous training of TB surveillance staff on proper data collection and entry, (iii) ensure that there are dedicate health information officers at sub-district and district levels, (iv) institute a web-based ETR.Net data collection portal to allow direct input at facility-level, and (v) implement a unique TB patient identifier to link information of TB patients who move between or transfer to other health facilities and/or sub-districts.

## Paper context

This study evaluated the TB electronic register surveillance system in Eden District, Western Cape Province, South Africa, using the Centers for Disease Control and Prevention’s (CDC’s) updated guidelines for evaluating public health surveillance systems. Our data shows that ETR.Net is a viable surveillance tool for Eden District and is well accepted by stakeholders involved in TB care and management. However, there is a need for robust data quality assurance mechanisms to improve data quality and the utility of ETR.Net system.
